# Reactive Oxygen Species Regulate the Levels of Dual Oxidase (Duox1-2) in Human Neuroblastoma Cells

**DOI:** 10.1371/journal.pone.0034405

**Published:** 2012-04-16

**Authors:** Simona Damiano, Roberta Fusco, Annalisa Morano, Mariarosaria De Mizio, Roberto Paternò, Antonella De Rosa, Rosa Spinelli, Stefano Amente, Rodolfo Frunzio, Paolo Mondola, Francoise Miot, Paolo Laccetti, Mariarosaria Santillo, Enrico Vittorio Avvedimento

**Affiliations:** 1 Dipartimento di Neuroscienze, Sezione di ­Fisiologia, Università degli Studi di Napoli “Federico II”, Napoli, Italia; 2 Dipartimento di Biologia Strutturale e Funzionale, Università degli Studi di Napoli “Federico II”, Napoli, Italia; 3 Dipartimento di Biologia e Patologia Molecolare e Cellulare, Istituto di Endocrinologia ed Oncologia Sperimentale del CNR, Università degli Studi di Napoli “Federico II”, Napoli, Italia; 4 Laboratorio di Ricerca Preclinica e Traslazionale, IRCCS, Centro di Riferimento Oncologico della Basilicata, Rionero in Vulture, Potenza, Italia; 5 Dipartimento di Medicina Clinica e Sperimentale, Università degli Studi di Napoli “Federico II”, Napoli, Italia; 6 Institut de Recherche Interdisciplinaire en Biologie Humaine et Moléculaire, Université Libre de Bruxelles, Brussels, Belgium; Sun Yat-sen University Medical School, China

## Abstract

Dual Oxidases (DUOX) 1 and 2 are efficiently expressed in thyroid, gut, lung and immune system. The function and the regulation of these enzymes in mammals are still largely unknown. We report here that DUOX 1 and 2 are expressed in human neuroblastoma SK-N-BE cells as well as in a human oligodendrocyte cell line (MO3-13) and in rat brain and they are induced by platelet derived growth factor (PDGF). The levels of DUOX 1 and 2 proteins and mRNAs are induced by reactive oxygen species (ROS) produced by the membrane NADPH oxidase. As to the mechanism, we find that PDGF stimulates membrane NADPH oxidase to produce ROS, which stabilize DUOX1 and 2 mRNAs and increases the levels of the proteins. Silencing of gp91*^phox^* (NOX2), or of the other membrane subunit of NADPH oxidase, p22*^phox^*, blocks PDGF induction of DUOX1 and 2. These data unravel a novel mechanism of regulation of DUOX enzymes by ROS and identify a circuitry linking NADPH oxidase activity to DUOX1 and 2 levels in neuroblastoma cells.

## Introduction

Dual oxidases or DUOX (the products of DUOX 1 and 2 genes) are NADPH oxidase (NOX) enzymes (EC 1.6.3.1) that contain the membrane-bound flavocytochrome segment of NADPH oxidase, fused to an extra-cellular peroxidase (EC 1.11.1) domain [1]-[4]. DUOX1 and 2 were first cloned in the thyroid but their expression has been so far demonstrated in several tissues, especially colon, lung and immune system [5]. Although these enzymes are important sources of local ROS, their function and regulation are still unknown. In thyroid DUOX proteins are localized to the apical pole of follicular cells, where they produce H_2_O_2_, necessary for organification of iodine and synthesis of thyroid hormones [6]-[8]. Targeted production of ROS by DUOX enzymes seems essential for gut immunity in Drosophila [9]. Since ROS production may be harmful to the tissue, DUOX enzymes are regulated by several positive and negative signals to finely adjust ROS production to the changing environment in lung or intestinal epithelium [10]. A positive stimulus is received by a not-yet identified G protein-coupled receptor, which signals through phospholipase C (PLC)-β to activate DUOX 1 and 2 expression; Ca^++^ ions, which are essential for their enzymatic activity, are able also to stimulate or inhibit DUOX 1 and 2 expression. As example, Ca^++^ activates calcineurin, which represses the transcription of DUOX 1 and 2 genes [11].

While targeted production of ROS is an essential element controlling immune homeostasis in gut, the function and the regulation of DUOX enzymes in other cell types, such as neurons, not directly exposed to the foreign environment, are not obvious. DUOX are also expressed in the respiratory tract epithelium and are differentially regulated by cytokines, but their specific function in this compartment remains still elusive [12].

ROS modulate different physiological functions, beside innate immunity, such as the biosynthesis of thyroid hormones, cellular signalling, gene expression, cellular growth and death [3], [13] **.** Oscillations of ROS levels mark proliferation and neoplastic transformation [14]. Inhibitors of oxidases (NOX/DUOX) appear to prevent in some circumstances complications in chronic diseases [15], [16].

ROS produced by NOX enzymes have been demonstrated to exert cell signalling functions in CNS modulating neuronal differentiation [17], synaptic activity [18], learning, memory and long term potentiation [19]-[21], etc. The presence of constitutive or inducible NOX1-4 isoforms have been widely demonstrated in CNS (for a review see [22]). Instead, the expression of DUOX enzymes in cells of CNS is not been clearly documented and no data are available about the regulation or functions of these enzymes in the CNS. The current proposed function of DUOX as component of peroxidase-host defence system, which is similar to the MPO-hydrogen peroxide in neutrophils, is easily applicable to lung, gut, thyroid, but difficult to extrapolate it to neurons.

To find the function of the DUOX system in neuronal cells, we have first investigated the presence of DUOX1 and 2 proteins in the brain and then the regulation of their expression in SK-N-BE cells. We have previously demonstrated that, in human neuroblastoma SK-N-BE cells, the Ras/extracellular signal-regulated kinase (ERK1/2) pathway stimulates NADPH oxidase inducing ROS levels [23]. By using a combination of biochemical and molecular techniques, we here demonstrate that DUOX 1 and 2 are rapidly induced by ROS produced by membrane NADPH oxidase. PDGF activates NADPH oxidase and increases ROS levels, which stabilize DUOX 1 and 2 mRNAs. These data reveal a regulatory network linking membrane NADPH oxidase, ROS and DUOX1 and 2 levels and highlight a novel function of DUOX enzymes in neurons as ROS sensors.

## Materials and Methods

### Ethics Statement

All animal procedures were approved by the Ethical Animal Care Committee of the University of Naples “Federico II” (prot. N. 2011/0082857 and prot. N. 2012/0009089).

### Reagents

Human recombinant PDGF-BB, apocynin, actinomycin D and 4-(2-Aminoethyl) benzenesulfonyl fluoride (AEBSF), were purchased from Sigma (USA). BAPTA-AM was purchased from Calbiochem (USA).

### Cell culture

Human neuroblastoma SK-N-BE cells (American Type Culture Collection, ATCC, USA) were grown in monolayer in RPMI 1640 medium supplemented with 10% foetal bovine serum (FBS), 2 mM L-glutamine, 50 μg/ml streptomycin and 50 IU/ml penicillin.

MO3.13 (CELLution Biosystem Inc., Canada), an immortal human-human hybrid cell line, expressing the molecular markers of oligodendrocytes, and Caco-2 cells, a human epithelial colorectal adenocarcinoma cells, were grown in Dulbecco’s Modified Eagles Medium (DMEM), containing 4.5g/L glucose, supplemented with 10% FBS, 100 U/ml penicillin and 100 μg/ml streptomycin. 

The cells were kept in a 5% CO_2_ and 95% air atmosphere at 37°C.

### Western blotting analysis

SK-N-BE and M03-13 cell lysates were obtained in RIPA buffer (50mM Tris-HCl, pH 7.5, 150mM NaCl, 1%NP40, 0.5% deoxycholate, 0.1% sodium dodecyl sulphate (SDS) containing 2.5mM Na-pyrophosphate, 1mM β-glycerophosphate, 1mM NaVO_4_, 1mM NaF, 0.5mM phenyl-methyl-sulfonyl-fluoride (PMSF), and a cocktail of protease inhibitors (Roche, USA). The cells were kept for 15min at 4°C and disrupted by repeated aspiration through a 21-gauge needle. Cell lysates were centrifuged for 10 min at 11,600xg and the pellets were discarded. Fifty micrograms of total proteins were subjected to sodium dodecyl sulphate – 7.5% polyacrylamide gel electrophoresis (SDS-PAGE) under reducing conditions. After electrophoresis, the proteins were transferred onto a nitrocellulose filter membrane (GE-Healthcare, UK) with a Trans-Blot Cell (Bio-Rad Laboratories, UK) and transfer buffer containing 25 mM Tris, 192 mM glycine, 20% methanol. Membranes were placed in 5% non-fat milk in phosphate-buffered saline, 0.5% Tween 20 (PBST) at 4°C for 2 hr to block the nonspecific binding sites. Filters were incubated with specific antibodies before being washed three times in PBST and then incubated with a peroxidase-conjugated secondary antibody (GE-Healthcare, UK). After washing with PBST, peroxidase activity was detected with the enhanced chemiluminescence (ECL) system (GE-Healthcare, UK).

DUOX 1 and 2 proteins were detected with a rabbit polyclonal antibody raised against the peptide sequence ETELTPQRLQC located inside the first intracellular loop of human DUOX1; the specificity of the antibodies was tested using human primary thyroid cells as positive control and pre-immune serum as negative control (not shown); goat anti gp91*^phox^* (NOX2) and rabbit p22*^phox^* or SOD1 polyclonal antibodies were purchased by Santa Cruz Biotechnology (USA);

The filters were also probed with an anti α-tubulin antibody (Sigma, USA). Protein bands were revealed by ECL and, when specified, quantified by densitometry using Scion Image software. Densitometric values were normalized to α-tubulin.

### Rat brain membrane preparation

Rat brain was homogenized in 250 mM sucrose, 5 mM imidazole, pH 6.5, and 0.5 mM dithiothreitol (1:4, wt/vol), using a glass-Teflon potter and then centrifuged at 800 *g* at 4°C for 10 min. The supernatant was centrifuged at 100,000 *g* at 4°C for 45 min in a 70.1 Ti rotor (Beckman, USA). The membrane pellet was suspended in RIPA buffer and 50 µg of proteins were then subjected to Western blotting.

### Real Time and semi-quantitative PCR

RNA isolation and real-time PCR were performed as follow: Total RNA was extracted using TRI-reagent according to the protocol provided by the manufacturer (Sigma, USA). Total RNA (4 µg) was reverse transcribed with Omniscript Reverse Transcriptase (Quiagen, USA) by oligo-dT primers for 60 min at 37°C in 40 µl reaction volumes. Real-time PCR was performed with an ABI 5700 or ABI PRISM-7900HT Sequence Detection System (Applied Biosystems Inc., USA). Reactions were carried out in 96-well optical reaction plates in a 50 µl final volume containing 25 µl of the SYBR-Green (Applied Biosystems Inc., USA) PCR master mix, 1,25 µl of each gene-specific primer, 40 ng of sample cDNA. Gene-specific primers were designed to selectively amplify DUOX1, DUOX2, DUOXA1 or NOX2 and relative expression values were normalized using glucose-6-phosphate dehydrogenase (G6PD). The SYBRGreen (Applied Biosystems Inc., USA) fluorescence was measured at each extension step. The threshold cycle (Ct) value reflects the cycle number at which the fluorescence measurement reached an arbitrary threshold. Melting curve analysis was performed to determine the specificity of the reaction. Real-time PCR was conducted in triplicate for each sample and the mean value was calculated.

Semi-quantitative PCR was performed in 20 µl final volume containing 0.5 µM of dNTP, 0.2 µM of the specific primers and 100 ng of sample cDNA. The PCR conditions used were 94°C 1.5 min, (94°C 30sec, 60°C 30 sec, 70°C 45 sec), 70°C 10 min. The reactions were carried out at different cycles (15–25–35).

Primers used in these studied are the following:

Human DUOX1: 5′-TTC ACG CAG CTC TGT GTC AA-3′ 3′-AGG GAC AGA TCA TAT CCT GGC T-5′

Human DUOX2: 5′- ACG CAG CTC TGT GTC AAA GGT-3′ 5′- TGA TGA ACG AGA CTC GAC CAG GC-3′.

Human G6PD (F), ACA GAG TGA GCCC TTC TTC AA (R), ATA GGA GTT GCG GGC AAA G

Human NOX1 (F), GTA CAA ATT CCA GTG TGC AGA CCA C (R), CAG ACT GGA ATA TCG GTG ACA GCA Human cytochrome b-245, beta polypeptide (CYBB, alias NOX2) (F), GGA GTT TCA AGA TGC GTG GAA ACT A (R), GCC AGA CTC AGA GTT GGA GAT GCT Human NOX 3 (F), CAC ACC ATG TTT TCA TCG TCT T **(R), GAA GAT ATG GCTGGG CAC TG**

Human NOX4 (F), GCT TAC CTC CGA GGA TCA CA (R), CGG GAG GGT GGG TAT CTA A Human NOX 5 (F), ATC AAG CGG CCC CCT TTT TTT CAC **( R), CTC ATT GTC ACA CTC CTC GAC AGC**

Human DUOXA1 (F), TTC ATC GTC ATC CTG CCT GGC ATT (R), TCC ACT CAG AAC TGA AGG CCT TGT

### RNA interference

Human NOX2 small interfering RNAs (siRNAs) were obtained from DHARMACON (ON-TARGETplus) (USA). Human p22*^phox^* siRNA (Stealth RNAi™) were obtained from Invitrogen Corporation (USA). Transfection of siRNAs was carried out by MicroPorator (MP-100) Digital Bio Technology, a pipette-type electroporation. Cells were dissociated by a brief treatment with trypsin-EDTA, and counted. Indicated plasmids, DNAs and siRNA were introduced into each 1X10^6^ dissociated cells in 300 µl volume according to manufacturer’s instructions. The experimental conditions were optimized for SK-N-BE cells: Voltage 1200, width 20 msec, 3 pulse. Electroporated cells were then seeded into culture dishes containing pre-warmed culture media.

We transfected independently 2 different NOX2 and p22*^phox^* siRNAs and obtained similar results. NOX2 or p22*^phox^* knockdown was tested by immunoblot. NOX2 knockdown was also tested by RT-PCR. As controls were used “nontargeting” (NT) scrambled siRNAs. In all experiments siRNAs were used at a final concentration of 100 nM and cotransfected with 2µg of Green Fluorescent Protein (GFP) plasmid to evaluate transfection efficiency. Percent of GFP positive cells were evaluated after 48h of transfection by flow cytometry. Transfection efficiency was 70 ±7%.

### Fluorimetric Determination of Reactive Oxygen Species

ROS levels were determined by the membrane-permeant ROS sensitive fluorogenic probe 5,6-carboxy-2’, 7’-dichlorofluoresceindiacetate, DCHF-DA (Molecular Probes, The Netherlands). SK-N-BE cells were grown to semi-confluence in 24 multiwell plates and incubated for 18h in medium containing 0.2% FBS before the experiments. The cells were washed twice with calcium-free PBS and incubated in the same buffer with 10µM DCHF-DA for 10min and with or without 10µM of the intracellular calcium chelator BAPTA-AM for 5 min at 37°C. Then, cells were washed three times with PBS containing 10mM glucose, 1.2 mM MgCl_2_ and 1.2 mM CaCl_2_ and stimulated with 15ng/ml of PDGF. Dichlorofluorescein (DCF) fluorescence was measured at different time intervals using the plate reader Fluoroskan Ascent FL fluorometer and data analysed by Ascent software.

### H_2_O_2_ measurement

Extracellular H_2_O_2_ levels were determined with the Colorimetric Hydrogen Peroxide kit from Stressgen (USA). 8x105 SK-N-BE cells were grown in 24- Multiwell plates and the test was carried out on 50µl of cell medium.

### Immunofluorescence and confocal microscope analysis of DUOX in rat brain sections

The animals were anesthetized intraperitoneally with chloral hydrate (200 mg/kg) and perfused transcardially with 4% paraformaldheyde in phosphate buffer. The brain was sectioned coronally at vibratome (slice thickness 100 µm) and after blocking in phosphate buffer containing 10% FBS, 1% BSA, 0.1% Triton X100 (buffer A) for 1h at room temperature, sections were incubated overnight at 4°C with a mix of primary goat anti human DUOX1 and DUOX2 antibodies from Santa Cruz Biotechnology (USA), at 1:100 dilution in buffer A. Then, the sections were washed in phosphate buffer, and incubated for 1h at room temperature with Cy5-conjugated anti goat Ig secondary antibody at 1:200 dilution in buffer A.

The sections were examined using a Zeiss LSM 510 Meta laser scanning confocal microscope. The negative control, incubated with secondary antibody alone, did not show any non-specific staining.

### Statistical analysis

Statistical differences were evaluated using a Student's *t*-test for unpaired samples.

## Results

### DUOX proteins are expressed in the brain

**Figure 1:**
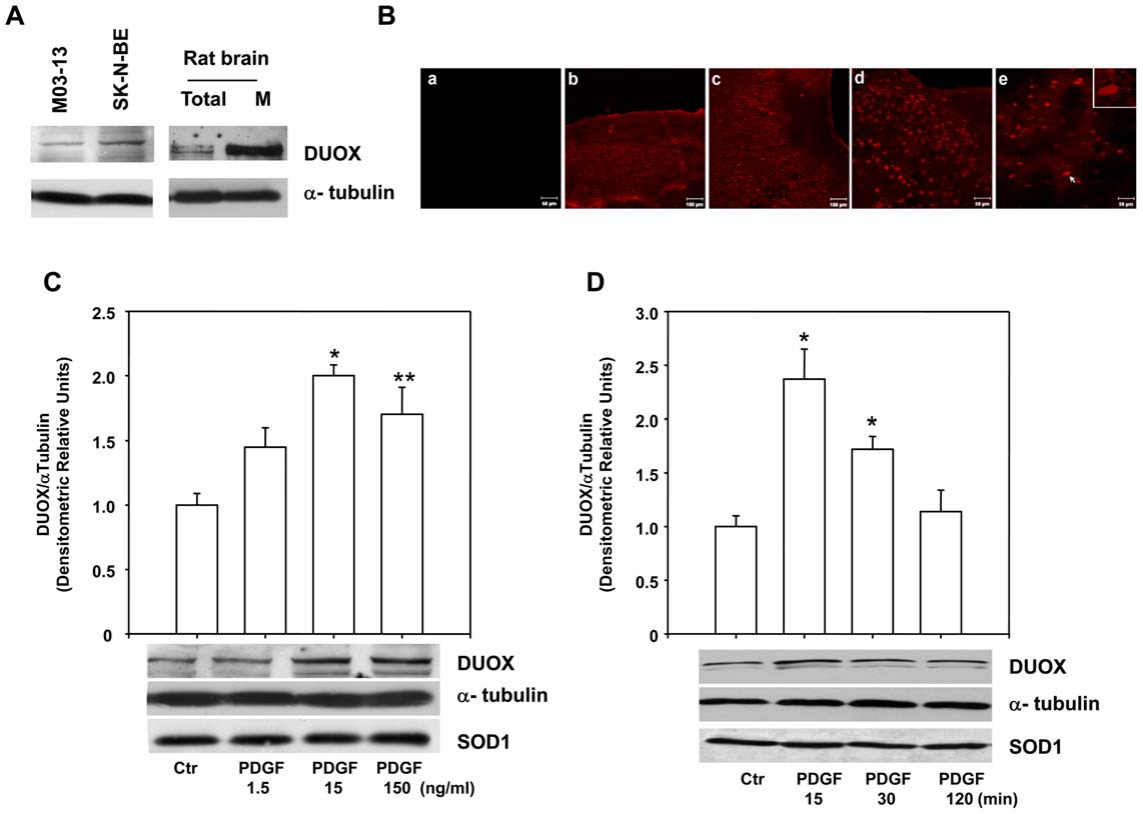
DUOX proteins are expressed in brain and are induced by PDGF in SK-N-BE cells. (**A**) Western blot analysis of DUOX proteins in the human oligodendrocyte cell line M03-13, human neuroblastoma (SK-N-BE) cells and rat brain total extract (total) and membrane fraction (M). (**B**) Confocal microscopic images displaying DUOX immunoreactivity in coronal rat brain slices. The negative control incubated with secondary antibody alone is shown in **a**; x10 (**b** and **c**) and x20 (**d** and **e**) magnifications are shown; in the inset of image **e** is shown a further magnification of the cell indicated by the white arrow to highlight the staining of both cell body and extensions. (**C**) SK-N-BE cells were incubated for 18h in medium containing 0.2% FBS and were then stimulated with increasing doses of PDGF for 15min before harvesting them for immunoblot analysis for DUOX protein. (**D**) SK-N-BE cells were incubated for 18h in medium containing 0.2% FBS and were then stimulated with 15ng/ml of PDGF for the indicated times before harvesting them for immunoblot analysis for DUOX protein. The histograms show the mean +/- SEM values relative to control obtained by densitometric analysis of DUOX bands normalized for α-tubulin of three independent experiments. The same blots were also probed with antibodies directed against superoxide dismutase (SOD) 1 enzyme to point out the specificity of the effect of PDGF on DUOX proteins. * p<0.01 and ** p<0.05 vs Ctr.

Since the presence of DUOX enzymes in the brain has not been firmly demonstrated, we first have analyzed by Western blotting the expression of DUOX1 and 2 proteins in rat brain, in a stabilized human oligodendrocytes cell line (M03-13) and in neuroblastoma (SK-N-BE) cells (Fig. 1A). In addition, to obtain information on the localization of these proteins at cell level, we have evaluated their presence in the membrane fraction of rat brain tissue. Both cell lines tested and brain tissue express high levels of the proteins that are concentrated in membranes. A specific DUOX immunoreactivity in the brain has been detected also in rat coronal brain slices by confocal immunofluorescence analysis (Fig. 1B). Large brain areas, in part corresponding to cortical layers, are selectively immunostained for DUOX. Higher magnification shows that the signal is confined to the cell body and dendrites.

### PDGF stimulates DUOX1 and 2 levels by increasing ROS in neuroblastoma cells

To study the regulation of these proteins, we have stimulated SK-N-BE cells with PDGF, which exerts trophic effects on a variety of neurons [24]-[26]. PDGF induces DUOX proteins in cells preincubated for 18 hrs in medium containing 0.2% FBS in a dose- and time-dependent manner with a peak of activity at 15 ng/ml for 15 min (Fig. 1C and D). These conditions have been used for all subsequent experiments. Serum starvation does not exert any toxic effect as evidenced measuring cell death by propidium iodide staining and cytofluorimetric analysis; also, serum starvation strongly downregulates the phosphorilation levels of ERK1/2, a kinase downstream PDGFR (data not shown) that normally increases in stress conditions.

**Figure 2:**
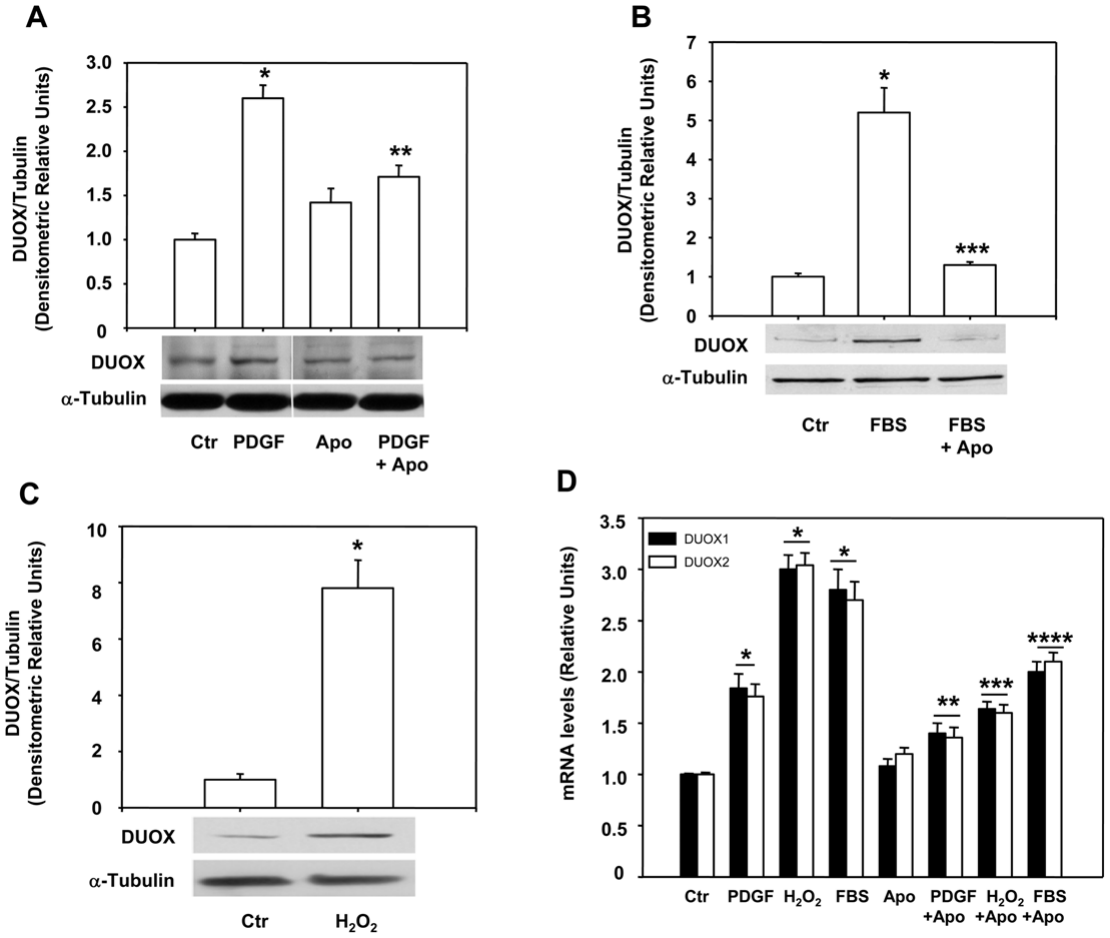
PDGF induction of DUOX levels is mediated by ROS. SK-N-BE cells were incubated 18h in medium containing 0.2% FBS, preincubated 1h with 50µM Apocynin (Apo) and then stimulated with 15ng/ml of PDGF (A) or 10% FBS (B) for 15min before harvesting them for immunoblot analysis of DUOX. The histograms show the mean +/- SEM values relative to control obtained by densitometric analysis of DUOX bands normalized for α-tubulin of three independent experiments. * p< 0.01 vs Ctr; ** p< 0.05 vs PDGF (A); ***p< 0.01vs FBS (B); (C) SK-N-BE cells were incubated 18h in medium containing 0.2% FBS and were then stimulated with 15μM H_2_O_2_ 15min before harvesting them for immunoblot analysis of DUOX. The histograms show the mean +/- SEM values relative to control obtained by densitometric analysis of DUOX bands normalized for α-tubulin of three independent experiments. * p< 0.01 vs Ctr. (D) RT- PCR analysis of DUOX 1 and 2 mRNA levels of SK-N-BE cells incubated 18h in medium containing 0.2% FBS, preincubated 1h in the presence or absence of 50µM Apocynin (Apo) and then stimulated with 15ng/ml of PDGF, 15μM H_2_O_2_ or 10% FBS for 15min. The histograms show the mean +/- SEM values relative to control of three independent experiments. * p< 0.01 vs Ctr; ** p< 0.05 vs PDGF; *** p< 0.01 vs H_2_O_2_. **** p< 0.05 vs FBS.

**Figure 3:**
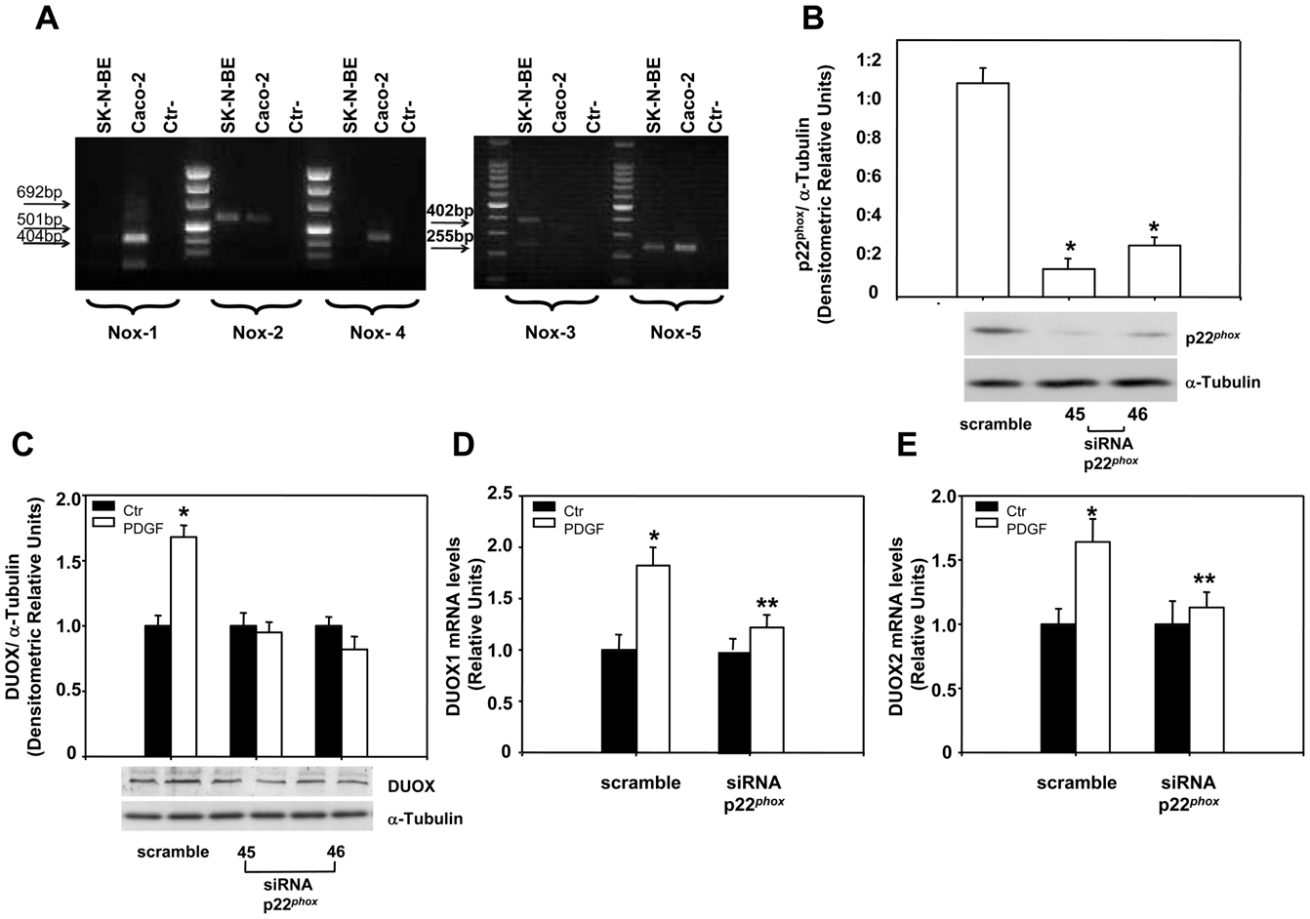
Silencing of p22*^phox^* inhibits the induction of DUOX 1 and 2 protein and mRNA levels by PDGF. (**A**) Identification of NOX expressed in neuroblastoma (SK-N-BE) and colon carcinoma (Caco-2) cells. Total RNA was extracted with Trizol, reverse transcribed and analyzed by PCR (see Materials and Methods) with specific primers to NOX1, NOX2 and NOX4 (left) or NOX3 and NOX5 (right). The number of cycles was 35. (**B**) Cells were transfected by electroporation with two different (45 and 46) siRNA to p22*^phox^* (siRNA p22*^phox^*) or control, scrambled siRNA (scramble) as described in Materials and Methods. 48h after transfection, total proteins were extracted and subjected to immunoblot analysis of p22*^phox^*. The histograms show the mean +/- SEM values relative to scramble obtained by densitometric analysis of p22*^phox^* bands normalized for α-tubulin of three independent experiments. *p< 0.01 vs scramble. (**C**) 24h after transfection cells were incubated in medium containing 0.2% FBS for 18h and then stimulated with 15ng/ml of PDGF for 15min. Total proteins were extracted and subjected to immunoblot analysis of DUOX. The histograms show the mean +/- SEM values relative to samples not stimulated with PDGF (Ctr) obtained by densitometric analysis of DUOX bands normalized for α-tubulin of three independent experiments. * p< 0.01 vs Ctr. (**D, E**) 24h after transfection with a mix of the two p22*^phox^* siRNA, cells were incubated in medium containing 0.2% FBS for 18h and then stimulated with 15ng/ml of PDGF for 15min, mRNA was extracted and DUOX1 and DUOX2 mRNA levels were analyzed by RT-PCR as described in Materials and Methods. The histograms show the mean +/- SEM values relative to samples not stimulated with PDGF (Ctr) of three independent experiments. * p<0.05 vs Ctr; ** p<0.05 vs PDGF stimulated scramble.

**Figure 4:**
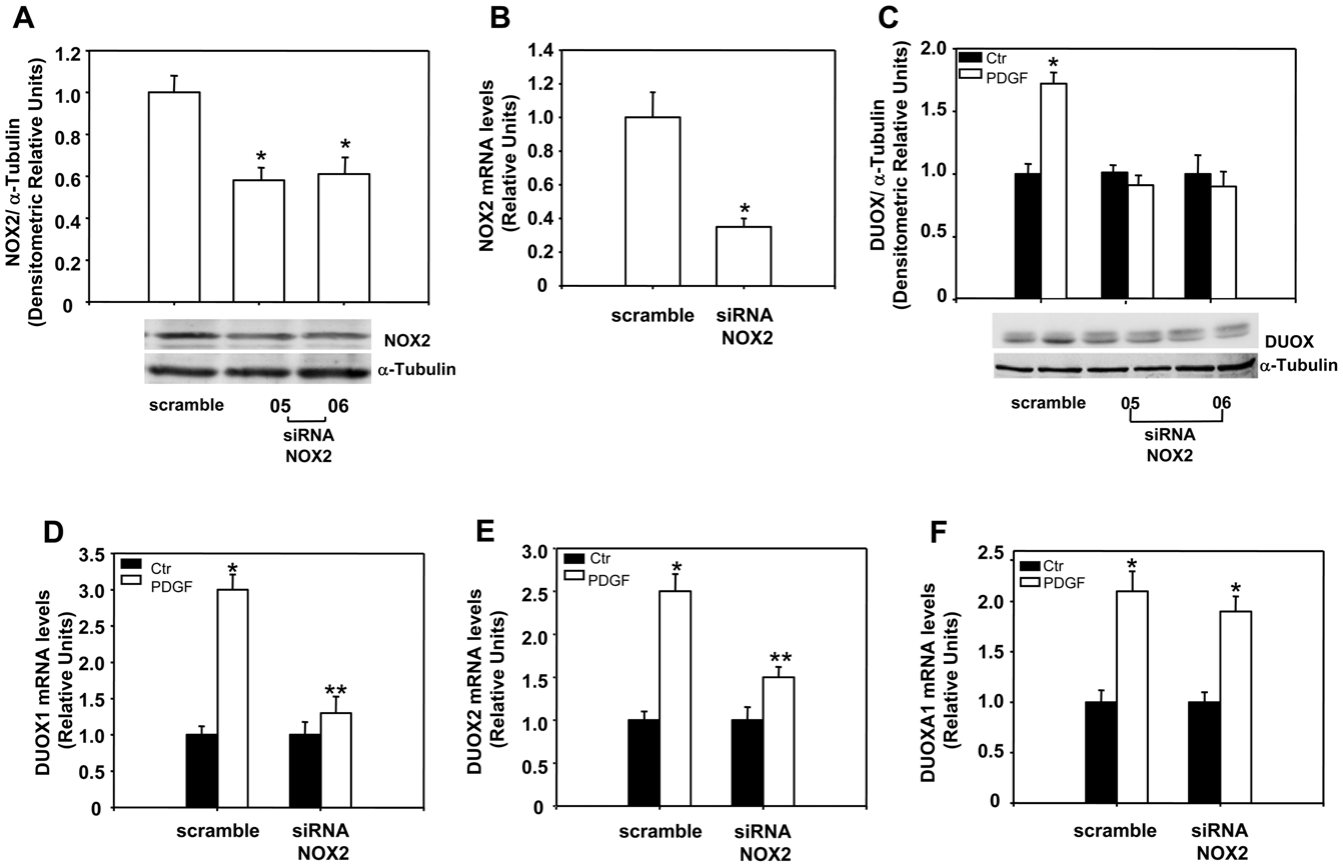
Silencing of NOX2 inhibits the induction of DUOX 1 and 2 protein and mRNA levels by PDGF. (**A-F**) SK-N-BE were transfected by electroporation with siRNA to NOX2 (05 and 06) (siRNA NOX2) or control, scrambled siRNA (scramble) as described in Materials and Methods. (**A**) 48h after transfection, total proteins were extracted and subjected to immunoblot analysis of NOX2. The histograms show the mean +/- SEM values relative to scramble obtained by densitometric analysis of NOX2 bands normalized for α-tubulin of three independent experiments. *p< 0.01 vs scramble. (**B**) 48h after transfection with a mix of the two NOX2 siRNA, mRNA was extracted and NOX2 mRNA levels were analyzed by RT-PCR as described in Materials and Methods. The histograms show the mean +/- SEM values relative to scramble of three independent experiments. * p< 0.01 vs scramble. (**C**) 24h after transfection cells were incubated in medium containing 0.2% FBS for 18h and then stimulated with 15ng/ml of PDGF for 15min. Total proteins were extracted and subjected to immunoblot analysis of DUOX. The histograms show the mean +/- SEM values relative to samples not stimulated with PDGF (Ctr) obtained by densitometric analysis of DUOX bands normalized for α-tubulin of three independent experiments. * p< 0.01 vs Ctr. (**D-F**) 24h after transfection with a mix of the two NOX2 siRNA, cells were incubated in medium containing 0.2% FBS for 18h and then stimulated with 15ng/ml of PDGF for 15min, mRNA was extracted and DUOX1 (**D**), DUOX2 (**E**) and DUOXA1 (**F**) mRNA levels were analyzed by RT-PCR as described in Materials and Methods. The histograms show the mean +/- SEM values relative to samples not stimulated with PDGF (Ctr) of three independent experiments. * p< 0.01 vs Ctr; **p< 0.01vs PDGF stimulated scramble.

Since PDGF stimulates in many cellular systems, neurons included, NADPH oxidase and increase ROS production [27] and (Agnese et al. in preparation), we have tested if ROS produced by membrane NADPH oxidase mediate PDGF effects on DUOX1 and 2 expression. To this end, we have treated the cells with PDGF or 10% FBS in the presence of apocynin, a ROS scavenger and inhibitor of NADPH oxidase [16]. Pre-treatment of cells with apocynin eliminates PDGF or 10% FBS induction of the protein (Fig. 2A and B) and mRNAs (Fig. 2D). The induction of DUOX 1 and 2 protein and mRNA levels by FBS indicates that growth factors other than PDGF could play a similar role in the regulation of the expression of these proteins. Also, hydrogen peroxide alone is able to induce DUOX protein and mRNA levels and this effect is eliminated by apocynin, an anti-oxidant an NADPH inhibitor [28] (Fig. 2C and D). These data indicate that ROS, induced by PDGF and other growth factors, stimulate DUOX1 and 2 expression (proteins and mRNAs); apocynin, as anti-oxidant and NADPH oxidase inhibitor [28], prevents PDGF, FBS or H_2_O_2_ induction of DUOX, suggesting that ROS, possibly generated by NADPH oxidase, mediate such an effect. To find the role of NADPH oxidase (NOX) on DUOX1-2 expression, we first determined which NOX is expressed in neuroblastoma cells. Fig. 3A shows that SK-N-BE cells express NOX2 and NOX5, as colon carcinoma cells (Caco-2) used as control [29] and NOX3 (Fig. 3A). Since the membrane subunit p22*^phox^* is closely associated with the catalytic membrane subunit of 4 of the 5 NOX isoforms (NOX1-4) [30], we have silenced this subunit to knock down all the NOX isoforms in SKB cells. Fig. 3B shows that the specific siRNAs selectively reduce p22*^phox^* protein levels and eliminate PDGF induction of DUOX enzymes (Fig. 3C). PDGF induction of DUOX 1 and 2 mRNA levels is also abolished by p22*^phox^* silencing (Fig. 3D and E). We have silenced also NOX2 (Fig. 4A and B) and we found that PDGF induction of DUOX protein (Fig. 4C) and mRNA levels (Fig. 4D and E) was abolished as well as in p22*^phox^* knock-down cells. The maturation factor DUOXA1, which is essential for the biological activity of the enzyme, is also stimulated by PDGF but its induction is not influenced by NOX2 levels (Fig. 4F). Taken together these data indicate that NOX2 is the major inducer of DUOX expression in neuroblastoma cells exposed to PDGF.

**Figure 5:**
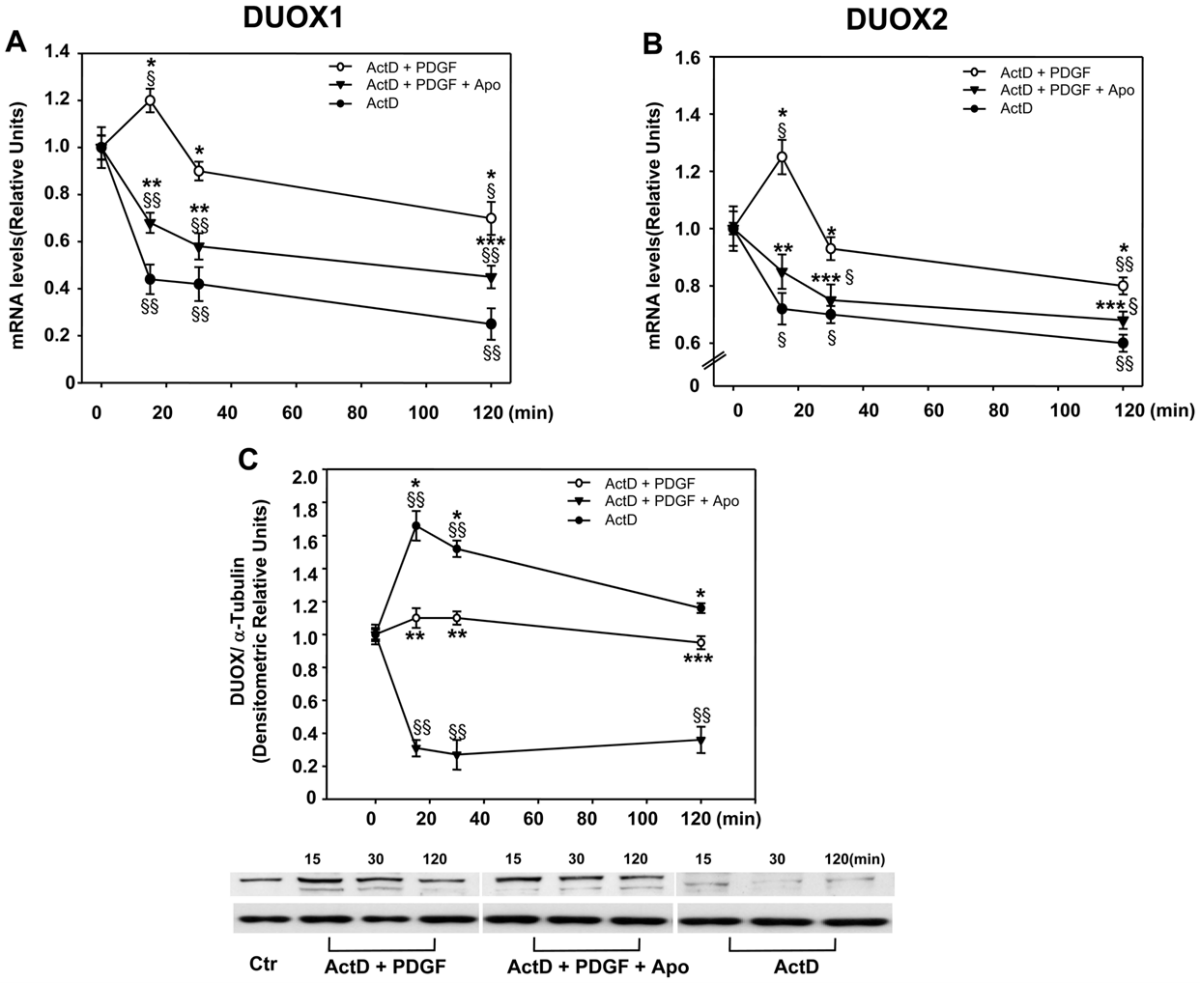
PDGF increases DUOX1 and 2 mRNAs stability. (**A, B**) RT- PCR analysis of DUOX 1 and 2 mRNA levels of SK-N-BE cells incubated 18h in medium containing 0.2% FBS, and then treated with of 1µg/ml of actinomycin D for the indicated times in absence or presence of 15ng/ml of PDGF and 50µM apocynin as indicated. Values are mean +/- SEM relative to control of three independent experiments. (**B**) Western blotting analysis of DUOX protein levels of SK-N-BE cells treated as in (**A**). Values are mean +/- SEM relative to control obtained by densitometric analysis of DUOX bands normalized for α-tubulin of three independent experiments. § p<0.05 and §§ p<0.01 vs Ctr; * p<0.01 vs the corresponding time point of ActD curve; ** p<0.01 and *** p<0.05 vs the corresponding time point of ActD + PDGF curve.

We next have examined whether the induction of DUOX1 and 2 mRNA levels by PDGF is mediated by stimulation of gene transcription or by a post-transcriptional mechanism. To test specifically this point, we have measured the half-life of DUOX1 and 2 mRNAs by exposing the cells for various periods of time to actinomycin D, under conditions of selective inhibition of RNA polymerase II-driven transcription. Fig. 5A and B show that the mRNAs of both proteins decay with a rapid kinetic with an half-life of 11min and 14 min for DUOX1 and DUOX2, respectively. PDGF is able to induce DUOX1-2 mRNA levels even in the presence of actinomycin D. In fact, the decay of the DUOX mRNAs was significantly modified by PDGF. The half-life of DUOX1 and DUOX2 mRNAs in cells exposed to PDGF is approximately 299 and 196 min, respectively, compared to 11 and 14 min, respectively in the absence of PDGF. This induction is mediated by ROS, because co-incubation of the cells with PDGF and apocynin in the presence of actinomycin D, eliminates the effects of PDGF on mRNAs stabilization by decreasing the half-life (18.5 and 22 min for DUOX1 and DUOX2, respectively). Under these conditions, apocynin inhibition of PDGF induction is reversed by hydrogen peroxide (not shown) supporting the hypothesis that ROS produced by NADPH oxidase are mediators of PDGF effects on mRNA stability. Western blotting analysis of DUOX protein levels measured in the same experimental conditions, shows that the time-dependent decay of the proteins in the presence of actinomycin D and PDGF is similar to that of mRNA levels and that apocynin inhibits PDGF induction of DUOX proteins. Collectively, these data indicate that PDGF regulates DUOX1-2 levels by modifying the stability of the specific mRNAs and that this effect is mediated by ROS generated by NADPH oxidases (Fig. 5C).

### PDGF stimulates both NOX and DUOX enzymes

**Figure 6:**
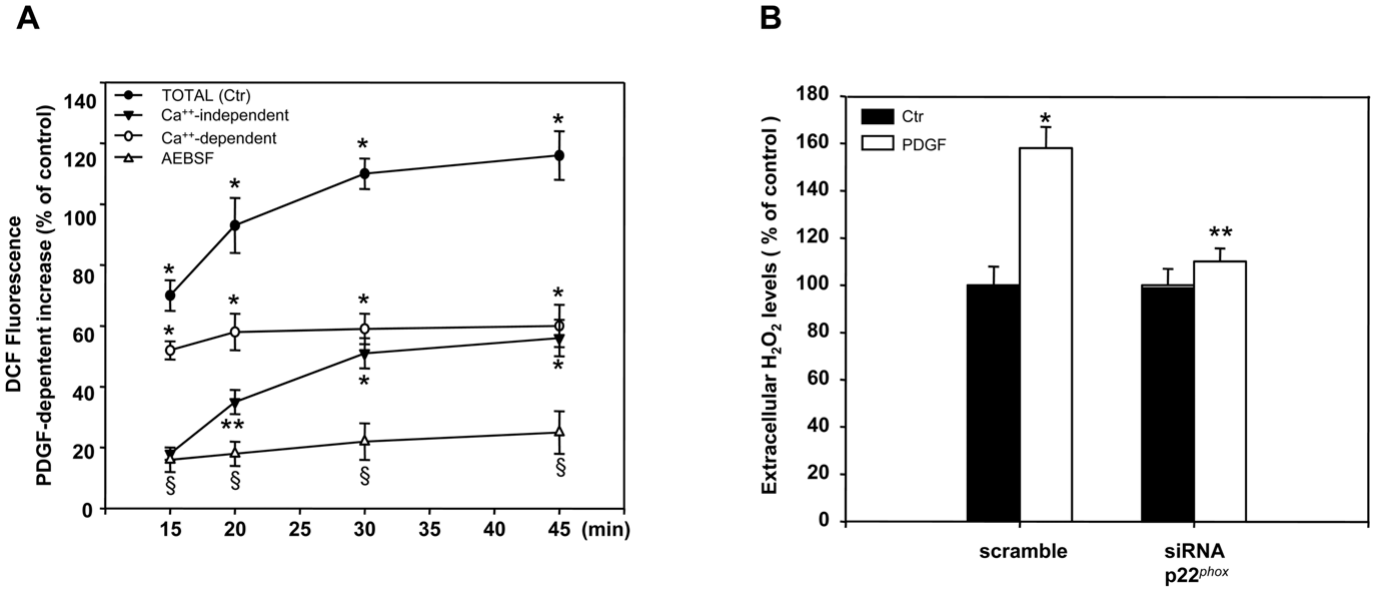
ROS levels in PDGF stimulated SK-N-BE cells. (**A**) Cells were incubated 18h in medium containing 0.2% FBS, loaded with 10µM DCHF-DA in the presence or absence of the intracellular calcium chelator, BAPTA-AM (10µM), and then stimulated with 15ng/ml of PDGF as described in Materials and Methods. ROS levels were measured fluorimetrically at the time intervals indicated. Ca^++^-independent ROS were measured in presence of BAPTA-AM. Ca^++^-dependent ROS levels were derived from the assays performed in the presence or absence of BAPTA-AM. Total levels of ROS induced by PDGF were also measured in the absence (Total) or presence of the NADPH oxidase inhibitor AEBSF. Values are Mean +/- SEM of three independent experiments performed in triplicate. * p< 0.01 and ** p< 0.05 vs not stimulated; § p<0.01 vs the corresponding time point of Total (Ctr) curve. (**B**) Cells were transfected by electroporation with siRNA to p22*^phox^* (siRNA p22*^phox^*) or control, scrambled siRNA (scramble) as described in Materials and Methods. 24h after transfection cells were incubated in medium containing 0.2%FBS for 18h and then stimulated with 15ng/ml of PDGF for 15min. An aliquot of cell medium was collected and analyzed for H_2_O_2_ levels as described in Materials and Methods. The histograms show the mean +/- SEM values of three independent experiments. * p< 0.01 vs Ctr. ** p< 0.01 vs PDGF stimulated scramble.

To test whether higher levels of DUOX proteins are translated into higher levels of enzymatic activity, we have measured ROS levels in the presence or absence of the intracellular calcium chelator BAPTA-AM. Since DUOX activity is tightly dependent on calcium, the difference between ROS levels measured in the presence and absence of BAPTA-AM represents an indirect measure of DUOX activity. We note, however, that also the activity of NOX5, expressed by neuroblastoma cells (Fig. 3A), is stimulated by calcium; NOX5 does not require p22*^phox^* subunit [30] and is not involved in the PDGF induction of ROS and DUOX1-2 expression, since silencing of p22 *^phox^* inhibits PDGF induction as well as silencing of NOX2 (Figs. 3, 4 and 6). Therefore, under our conditions, we believe that the major Ca-stimulated ROS producing activity induced by PDGF is the activity of DUOX enzymes. PDGF induces total ROS levels through NADPH oxidase activation as demonstrated by the inhibition of ROS induction by PDGF in the presence of NADPH oxidase inhibitor AEBSF (Fig. 6A). In addition, Fig.6A shows that a substantial fraction of PDGF stimulated-ROS is sensitive to BAPTA-AM and that calcium-resistant ROS (mainly produced by the other NOX enzymes) increase faster than the calcium-sensitive fraction (mainly DUOX-dependent), suggesting that NOX precedes DUOX activation. To test directly whether the induction of extracellular H_2_O_2_ levels by PDGF were mediated by NADPH oxidase activation, we measured extracellular H_2_O_2_ in cells, in which p22*^phox^* was silenced by the specific siRNA. In these cells PDGF was not able to induce extracellular H_2_O_2_ (Fig. 6B) as well as and DUOX1 and 2 expression (Fig. 3C, D and E).

Collectively, these data demonstrate that ROS produced by NADPH oxidase mediate PDGF induction of DUOX 1 and 2 expression by stabilizing the specific mRNAs.

## Discussion

### DUOX in brain

This is the first report demonstrating the presence of DUOX in the brain by immunofluorescent staining of rat brain slices or by immunoblot analysis of rat brain membrane and oligodendrocyte or neuroblastoma SK-N-BE cell line (Fig. 1). The function of DUOX enzymes in the nervous system and specifically in neuronal cells is not apparently linked to the innate immunity, as shown in other tissues. DUOX proteins are dual function enzymes, containing not only the ROS generating domain, homologous to NADPH enzymes but also a peroxidase domain, that may be used to carry on oxidations of other proteins. In C. Elegans, for example a similar Duox enzyme catalyzes the cross link of tyrosine residues in the cuticle (for a review see [1]). It is possible that a ROS-regulated activity of DUOX enzyme may be important in neurons to modify membrane proteins during synapses formation or maintenance.

### PDGF stimulates DUOX 1 and 2 expression

The data reported here indicate that PDGF and serum are able to induce rapidly DUOX1 and 2 in neuroblastoma cells (Fig. 1 and 2). PDGF induces a rapid (15–30 min) post-transcriptional stabilization of the DUOX1 and 2 specific mRNAs (Fig. 5), by stimulating the membrane NADPH oxidase, which appears to be the first target of PDGFR action. Thus, PDGF induction of DUOX1 and 2 is abolished by silencing p22*^phox^* or NOX2 (Fig. 3 and 4). Our data indicate that DUOX mRNAs are very unstable in the absence of ROS and consequently also the levels of the protein are low under these conditions. Stimulation of the cells by growth factors or microrganisms can induce a strong cellular response by increasing the levels of DUOX proteins on the membranes.

We suggest the following link between PDGF and DUOX: PDGF and its receptor binds NOX2 (Agnese et al., in preparation), induces ERK 1/2 and phosphoinositide 3-kinase (PI3K), which in turn activate NADPH oxidase [31], [27], [32]. ROS, produced by NADPH oxidase, inhibit tyrosine phsophatases and maintain active ERK1/2 [32]. Ultimately, ERK1/2 may stabilize the specific DUOX1-2 mRNAs and increase the levels of the proteins [33], [34].

### DUOX1 and 2 are exquisite sensors of ROS in neurons

**Figure 7:**
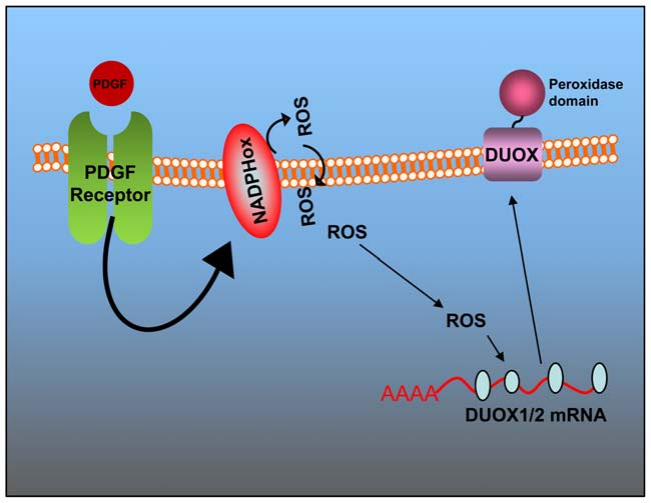
Regulation of DUOX by PDGF receptor. Schematic diagram showing the circuitry induced by PDGF leading to DUOX induction. PDGF activates the specific receptor, which stimulate NADPH oxidase. This sets off a loop that amplifies ROS cascade [32], [38]. ROS stabilize DUOX mRNAs and increase the level of DUOX proteins in plasma membrane.

Our data indicate that DUOX 1 and 2 mRNAs are rapidly degraded under normal conditions and accumulate upon oxidative stress (Fig. 5). Following PDGF stimulation, cellular ROS increase and this is accompanied by rise of DUOX enzymes (Fig. 2 and 6). We do not know the specific function of DUOX in neurons but it is possible that targeted DUOX protein focus ROS production in a specific membrane compartment contacting the extra-pericellular space (Fig. 7). In other cell types, such as thyroid cells or leukocytes, this is relevant for the formation of the iodine fixation complex on the apical membrane of thyroid cells or the formation of the spatial tissue gradient of hydrogen peroxide, required for rapid recruitment of leukocytes to the wound [35].

### The function of DUOX enzymes: focusing and targeting ROS production to the membrane

The regulation of DUOX expression by ROS seems conserved in different cell types. In lymphocytes, calcium and ROS generate a loop that modulates the strength of the final signal triggered by the receptor(s) and DUOX activity is central in this circuitry [36]. ROS production, upon DUOX induction, becomes restricted to discrete membrane segments. Apparently, this function cannot be complemented by membrane NADPH oxidase and has been exploited by intestinal epithelium for maintenance of gut immunity [11], by thyroid cells for iodine organification [37] and by lymphocytes for signaling [36]. It remains to be seen the function of DUOX in neurons and oligodendrocytes. Highly compartmentalized ROS production coupled to peroxidase activity may induce membrane fusion by cross-linking membrane proteins. We believe and suggest that this may be relevant in neurons for the formation and maintenance of synapses.
